# Validation of the Parental Playfulness Questionnaire in a Russian sample: development of a unidimensional 8-item short form (PPQ-8) and its association with parental stress

**DOI:** 10.3389/fpsyg.2026.1886533

**Published:** 2026-08-04

**Authors:** Vera L. Sukhikh, Margarita N. Gavrilova

**Affiliations:** 1 Federal Scientific Centre for Psychological and Multidisciplinary Research, Moscow, Russia; 2 Department of Psychology of Education and Pedagogy, Faculty of Psychology, Lomonosov State Moscow University, Moscow, Russia

**Keywords:** cross-cultural adaptation, parent practices, parental playfulness, parental satisfaction, parental stress, parental warmth, parent–child interaction, psychometric validation

## Abstract

**Introduction:**

Parental playfulness is increasingly recognized as a positive relational resource supporting child development and buffering parental stress. The Parental Playfulness Questionnaire (PPQ) is the only brief self-report instrument tailored to this construct; however, it has not undergone formal cross-cultural psychometric revalidation outside its original language and cultural context.

**Methods:**

The present study evaluated the Russian adaptation of the PPQ and its association with parental stress in a sample of 889 parents of children aged 2–7 years. Random split-half exploratory (*n* = 444) and confirmatory (*n* = 445) subsamples were used to test the factor structure of the instrument.

**Results:**

The original five-factor structure did not replicate: four of five subscales showed unacceptable reliability. A systematic method effect emerged, associated with content-reversed items and likely amplified by the cultural endorsement of involved parenting as a normative imperative. After removing reverse-scored items and items lacking cultural equivalence, an 8-item unidimensional short form (PPQ-8) demonstrated good model fit (CFI = 0.976, RMSEA = 0.066, SRMR = 0.054) and acceptable internal consistency (*α* = 0.784). The PPQ-8 was positively related to parental warmth and structure, and negatively to parental stress, supporting its external validity.

**Discussion:**

Considerable deviations between the final solution and the original five-factor structure suggest that the PPQ-8 may function more as a revised instrument than a mere abbreviation. The PPQ-8 offers an efficient measure of active parental playfulness and represents an important step toward a more comprehensive, culturally informed understanding of parent–child relationships.

## Introduction

Parenting practices encompass a broad spectrum of behaviors, attitudes, and emotional exchanges that shape children’s cognitive, social, and emotional development (Bornstein, 2022; Budagova, 2025). Among these practices, the quality of parent–child interaction has been consistently identified as a critical determinant of child well-being. Parental stress and parental playfulness as two key dimensions of parenting have emerged as critical factors influencing interaction quality and child outcomes. Parental stress, characterized by aversive psychological and physiological reactions to parenting demands, can compromise parents’ capacity for sensitive, responsive caregiving (Ward and Lee, 2020). Cross-cultural research further indicates that parental psychological resources such as self-efficacy and grit play a critical role in helping caregivers cope with parenting demands (Wahidah et al., 2025). Conversely, parental playfulness, defined as parents’ ability to engage spontaneously and joyfully with their children in play, and in other parent–child interactions, represents a positive relational quality that enhances parent–child bonding and supports child development (Wu et al., 2024; Deans, 2020).

Research distinguishes parental playfulness from general parenting behaviors such as sensitivity or structuring, though these dimensions are interrelated. Playfulness specifically captures the spontaneous and creative aspects of parent–child engagement that characterize high-quality play interactions (Seal et al., 2025).

A growing body of empirical work demonstrates that parental playfulness is associated with a wide range of positive child outcomes. Menashe-Grinberg and Atzaba-Poria (2017) showed that parental playfulness moderates the link between parental behavior and child negativity during play interactions, while Shorer et al. (2021, 2022, 2023, 2025) have repeatedly demonstrated its role in supporting children’s emotion regulation, reducing stress reactions during the COVID-19 outbreak, and mediating associations between parents’ emotional difficulties and children’s psychosocial adjustment. Recent studies further suggest that playful parents tend to raise playful children (Wu et al., 2024; Wei et al., 2025), and that parental playfulness contributes to the development of playfulness in children with low executive functions (Duss et al., 2024). Parental playfulness has also been linked to parenting styles, coparenting quality, and the broader family climate (Seal et al., 2025; Korgozha, 2024).

Emotion regulation theories emphasize that parents’ capacity to regulate their own emotions influences their ability to engage playfully with children (Shorer et al., 2021). When stress impairs parental emotion regulation, it may reduce parents’ psychological availability for joyful play. Conversely, playful engagement may serve as an emotion regulation strategy that helps parents manage stress.

Developmental systems perspectives highlight the transactional nature of parent–child relationships, wherein parental characteristics (including stress and playfulness) and child characteristics mutually influence each other over time (Schneider et al., 2022). Within this framework, parental playfulness may moderate the impact of stress on parent–child interaction quality and child outcomes.

Understanding the relationship between these constructs is essential for developing effective family interventions, particularly given the increasing recognition that parental playfulness may buffer against stress-related impairments in parenting quality.

Despite this accumulating evidence, the assessment of parental playfulness remains methodologically limited, with most studies relying on observational coding or adult playfulness scales not specifically designed for the parenting context. The Parental Playfulness Questionnaire (PPQ), developed by Shorer and colleagues, addresses this gap by offering a brief self-report tool tailored to capture playfulness within the parent–child relationship (Shorer et al., 2021). To date, the PPQ has been used in several studies beyond its original validation, with reported internal consistency ranging from approximately *α* = 0.67 to α = 0.84 across samples (Shorer and Leibovich, 2022; Shorer et al., 2021, 2025; Seal et al., 2025). However, no published forward–backward translation procedure, formal cultural adaptation, or independent psychometric revalidation (EFA/CFA, test–retest, item-response analyses) of the instrument in another language or cultural context appears to be available. This gap is particularly notable because playfulness, humor, and parent–child interaction styles are known to vary substantially across cultures. Robust cross-cultural psychometric evidence is therefore essential for the meaningful use and comparison of the PPQ across different samples.

Russian parenting culture has traditionally emphasized responsibility, moral upbringing, and structured developmental activities, while spontaneous and improvisational play has historically held a less prominent position in normative parental scripts (Karabanova, 2005; Polivanova et al., 2024; Rudnova et al., 2024; Sobkin et al., 2024). At the same time, contemporary Russian parents are increasingly influenced by global ideals of positive, child-centered parenting. This dual normative landscape may affect how individual PPQ items are interpreted and endorsed. Examining the PPQ in a Russian sample thus provides not only a practical adaptation but also a meaningful test of the instrument’s cross-cultural robustness.

The present study therefore pursues two interrelated aims: (1) to evaluate the psychometric properties of the Russian adaptation of the Parental Playfulness Questionnaire, including its factor structure, reliability, and if needed, derive a culturally adequate form; and (2) to examine the relationship between parental playfulness and parental stress in a Russian sample. We hypothesized that the Russian version of the PPQ would replicate the factor structure and demonstrate adequate reliability reported in the original validation studies, and that higher levels of parental playfulness would be associated with lower levels of parental stress.

## Materials and methods

### Sample

The study sample comprised 889 parents (858 mothers and 31 fathers) raising children aged 2 to 7 years (M = 54.19; SD = 15.61 months); (460 girls and 429 boys). The respondents permanently reside in the Russian Federation, in both large cities and rural areas. Of the sampled parents, 598 (67%) had more than one child. Half of the respondents were parents aged 18 to 35 years (51%), and 43.6% were aged 35 to 45 years. 64% of the participants held a higher education degree, 27% had completed specialized secondary education, and 9% had completed secondary education. Respondents reported their household income level as follows: 37.1% indicated that their income was sufficient for all needs except major purchases (e.g., an apartment or a country house); 26.4% could afford household appliances but not a car; and 24.4% could afford clothing and footwear, but purchasing household appliances would be difficult.

The achieved sample size (*N* = 889) substantially exceeded the conventional recommendations for factor-analytic studies (i.e., N ≥ 300 or at least 10 respondents per item; Comrey and Lee, 1992) and provided more than adequate statistical power (>0.99) to detect small correlations (r ≥ 0.10) at *α* = 0.05.

### Procedure

Data were collected in July–August 2025. Invitations to participate in the survey were distributed via email through municipal educational organizations. The email informed recipients of the opportunity to participate in a survey on parental beliefs about child-rearing and provided all necessary information about the study. Parents were then invited to follow a link to complete an online questionnaire. Upon obtaining informed consent from each participant, the survey was launched on the platform with a restriction preventing repeated submissions from the same device. Participation was voluntary and anonymous, no personally identifying information was collected, and no compensation was provided to respondents. The study and consent procedures were approved by the Research Ethics Committee of the Federal Scientific Center of Psychological and Multidisciplinary Research (decision of January 31, 2024, No. 3).

### Measures

The original *Parental Playfulness Questionnaire* (Shorer et al., 2021) consists of 20 items rated on a 5-point Likert scale and comprises five theoretically derived subscales: Spontaneity (6 items, e.g., enjoying spontaneous activities with the child), Enjoyment as a Problem-Solving Strategy (4 items, e.g., using games and humor to resolve parenting conflicts), Creativity and Improvisation (5 items, e.g., improvising during shared reading), Enjoyment as a Means of Emotion Regulation (3 items, e.g., using playful activities to soothe the child), and Cognitive Flexibility (2 items, e.g., finding creative compromises in conflict situations); in the original validation study, internal consistency for the total scale was *α* = 0.82 (Shorer et al., 2021).

The adaptation followed a committee approach (Van de Vijver and Tanzer, 2004), in which conceptual and cultural equivalence is established through iterative collaboration among a professional translator, content experts, and target respondents rather than through back-translation. This approach is recognized as an acceptable adaptation strategy, particularly when items require cultural rather than purely linguistic adjustment (Muñiz et al., 2013). The procedure comprised four steps: (1) forward translation by a professional translator familiar with both cultures; (2) expert review by the research team; (3) cognitive pretesting with parents (*n* = 17), who completed the questionnaire and provided structured feedback; and (4) iterative revision until conceptual equivalence was reached. Back-translation was deemed less suitable because several items (e.g., Halloween-related content, “power struggles”) required functional rather than literal re-expression (Hambleton and Zenisky, 2011).

Based on the combined input from expert review and pilot feedback, three items were flagged as potentially problematic for the Russian context. Item 11 referred to brainstorming Halloween costume ideas – a culturally specific practice with no direct equivalent in the Russian family calendar; the item was therefore reformulated to refer to New Year and other costume-based celebrations, which represent the closest functional analogue in Russian culture. Item 18 in the original version referred to a situation in which a child receives an open-ended assignment at school or preschool. Such situations are relatively uncommon in the Russian educational system, where structured tasks following clear instructions or templates traditionally predominate. As a result, many respondents perceived the original situation as hypothetical rather than as a familiar parenting context. Item 19, which framed parent–child interactions in terms of “power struggles”, posed particular difficulty: this construction is rarely used in Russian to describe family relationships and tends to be associated with political or organizational contexts. The item was therefore rephrased to convey the underlying notion of conflicting wishes between parent and child while avoiding the literal translation of “power”. These items were discussed and revised by the research team until conceptual equivalence with the original items was reached. The final Russian version preserved the structure, item sequence, instructions, and response scale of the original instrument.

To evaluate the external validity of the questionnaire under study, self-report measures previously adapted for Russian samples were employed.

*The Parental Stress Scale* (Berry and Jones, 1995; Russian adaptation by Bochaver et al., 2024) comprises 16 items rated on a Likert scale from 1 (strongly disagree) to 4 (strongly agree), distributed across two subscales: Parental Stress and Parental Satisfaction. Parental playfulness and parental stress represent conceptually distinct constructs pertaining to different aspects of parental functioning. The PPQ measures a behavioral characteristic – the propensity for spontaneous, flexible, and creative behavior in parent–child interactions (Shorer et al., 2021). The Parental Stress and Parental Satisfaction subscales, in contrast, reflect an affective-evaluative characteristic – the subjective experience of being in the parental role (Bochaver et al., 2024; Abidin and Wilfong, 1989). Although more playful parents may experience less stress and greater satisfaction due to adaptive coping strategies (Magnuson and Barnett, 2013), playfulness cannot be reduced to the mere absence of stress.

In *the Item-reduced Comprehensive General Parenting Questionnaire* (Sleddens et al., 2014; Russian adaptation by Kornienko and Rudnova, 2023) respondents were asked to indicate their agreement with each item on a scale from 1 (strongly disagree) to 5 (strongly agree). To reduce respondent burden and minimize attrition during survey completion, only items pertaining to the structural aspects of parenting were included. These items concern helping the child with tasks, organizing the child’s daily life, displaying responsiveness as an aspect of nurturance, and monitoring as an aspect of behavioral control (Kornienko and Rudnova, 2023).

*The Parenting Practices Questionnaire* (Cumming et al., 2022) provided the second block of items designed to measure warmth and emotional closeness (reliability of 0.73 in a Russian sample; Kornienko et al., 2024). This block includes four items describing various emotional expressions on the part of the parent (e.g., “Even when I am not in the mood, I show my child that I love him/her very much”, “The time my child and I spend together is filled with warmth and closeness”). Each item was rated by respondents on a scale from 1 (strongly disagree) to 4 (strongly agree). Parental warmth, as a behavioral characteristic, reflects emotional involvement, positive affect, and acceptance in the parent–child relationship. Parental playfulness is theoretically considered to be one manifestation of a positive, emotionally warm parenting style.

### Data analysis

To avoid capitalization on chance when both extracting and confirming the factor structure, the total sample (*N* = 889) was randomly split into two approximately equal subsamples: an exploratory subsample (*n* = 444) used for item analysis, parallel analysis, and exploratory factor analysis (EFA), and a confirmatory subsample (*n* = 445) reserved for confirmatory factor analysis (CFA), reliability, and validity testing. Random assignment was performed prior to any analysis, and identical distributions of child age, child gender, and parental education across the two subsamples were verified.

All analyses were conducted in JASP (version 0.19) with missing data handled according to the program’s default settings, namely pairwise exclusion in exploratory factor analysis and listwise exclusion in confirmatory factor analysis and correlation analyses.

## Results

### Internal consistency of the questionnaire scales

Descriptive statistics of the questionnaire items revealed non-compliance with the normal distribution assumption according to the Shapiro–Wilk test and severe violations of normality (Finney and DiStefano, 2006), as well as ceiling effects for items 14. “When we go to new places like the zoo or an amusement park, I like to watch my child explore and encourage him/her to ask questions” and 15. “I think being silly with my child is a waste of time” (Table 1). All Shapiro–Wilk tests were significant at *p* < 0.001.

**Table 1:** Descriptive and reliability statistics for the PPQ (*N* = 889).

Item	M	SD	Skewness	Kurtosis	Item-rest correlation r (by subscale)	Cronbach’s α if item dropped (by subscale)	McDonald’s ω if item dropped (by subscale)
Spontaneity							
3 *	2.41	1.06	0.289	−0.509	0.285	0.450	0.501
6 *	2.67	1.14	0.193	−0.640	0.402	0.386	0.445
8 *	2.46	1.08	0.397	−0.368	0.367	0.409	0.484
12 *	2.96	1.24	0.071	−0.852	0.352	0.410	0.497
17	3.60	1.20	−0.486	−0.611	−0.094	0.632	0.636
18 *	3.58	1.12	−0.400	−0.501	0.330	0.426	0.504
Enjoyment as a problem-solving strategy							
1	4.22	0.859	−0.945	0.612	0.396	0.644	0.652
2	4.15	0.928	−1.070	1.01	0.479	0.594	0.609
4	3.86	1.02	−0.634	−0.104	0.513	0.567	0.579
5	3.69	1.19	−0.495	−0.746	0.453	0.618	0.623
Creativity and improvisation							
7	4.07	0.974	−0.904	0.344	0.303	0.376	0.420
9	3.92	1.21	−0.966	−0.049	0.296	0.374	0.455
11	3.38	1.38	−0.326	−1.120	0.275	0.400	0.461
14	4.72	0.609	−2.430	6.76	0.334	0.397	0.413
15 *	4.51	0.943	−2.130	4.1	0.112	0.492	0.519
Enjoyment as a means of emotion regulation							
13	4.27	0.83	−1.080	1.05	0.224	0.048	0.048
16	3.60	1.15	−0.478	−0.412	0.210	−0.005	−0.001
20	3.49	1.21	−0.386	−0.723	0.016	0.465	0.484
Cognitive flexibility							
10 *	3.34	1.15	−0.113	−0.759	−0.029	—	—
19	3.83	1.09	−0.748	0.018	−0.029	—	—

*Reverse-scored item.

Reliability estimates are reported below for the total scale and each of the five subscales; item-level statistics (means, standard deviations, item-rest correlations, *α*-if-item-deleted, *ω*-if-item-deleted) are presented in Table 1.

The total scale demonstrated marginal internal consistency (Cronbach’s *α* = 0.688, McDonald’s *ω* = 0.728). At the item level, six predominantly reverse-scored items exhibited item-rest correlations below the conventional 0.20 threshold (items 3, 6, 8, 12, 18, and 19), indicating poor discrimination within the total scale; item 3 showed a negative correlation (r = −0.129), and its removal would increase α to 0.713. At the subscale level, Enjoyment as a Problem-Solving Strategy demonstrated the highest internal consistency among others (α = 0.673, ω = 0.681), although it still fell below the conventional 0.70 threshold (Nunnally and Bernstein, 1994). The remaining four subscales exhibited unacceptable internal consistency. For Spontaneity (α = 0.507, ω = 0.556), the only direct item (item 17) correlated negatively with the rest of the subscale (r = −0.094). For Creativity and Improvisation (α = 0.466, ω = 0.505), the reverse-scored item 15 showed near-zero discrimination (r = 0.112). For Enjoyment as a Means of Emotion Regulation (α = 0.255, ω = 0.483), item 13 was virtually unrelated to the other two items (r = 0.016). Finally, for Cognitive Flexibility, the two constituent items were essentially uncorrelated (r = −0.029, α = −0.060), rendering the subscale statistically non-functional; McDonald’s ω could not be meaningfully estimated for this two-item subscale. A consistent pattern emerged across all analyses. Reverse-scored items had lower means compared to direct items and tended to cluster together rather than with their assigned subscales, suggesting a “method effect” associated with content-reversed items (Weijters et al., 2013). These findings indicate that the original five-factor structure of the PPQ does not replicate at the reliability level in the Russian-language sample, warranting exploratory factor analysis to identify an empirically supported dimensional structure.

### Exploratory factor analysis

The exploratory subsample (*n* = 444) was used to identify a parsimonious factor structure. Given the ordinal scale and pronounced ceiling effects on directly worded items, all analyses relied on polychoric correlations and minimum residuals extraction, with oblimin rotation when more than one factor was retained.

An initial EFA on all 20 items reproduced the artifactual pattern previously documented for mixed-keyed instruments (Weijters et al., 2013). The three-factor solution suggested by parallel analysis separated directly worded items, items with ceiling effects, and reverse-scored items into distinct factors, with a latent correlation of only r = −0.24 between the “direct” and “reverse” factors. Fit was unsatisfactory (CFI = 0.869, TLI = 0.812, RMSEA = 0.073 [0.066, 0.080], SRMR = 0.045) and only 36.5% of variance was explained. Because the multifactor structure reflected item wording rather than substantive content, all seven reverse-scored items were excluded.

A single-factor EFA on the 13 directly worded items led to sequential item removal based on standardized loadings < 0.50, uniqueness > 0.75, and substantive considerations. Items 19 and 20 failed to load on the common factor (loadings < 0.35); Item 19 had been flagged during translation, as “power struggles” lacks a culturally equivalent rendering in Russian (Van de Vijver and Tanzer, 2004). Item 17 was removed due to limited contribution and redundancy with better-performing items. Item 11 (Halloween costume preparation, translated as costume parties — a non-salient tradition in Russia) and Item 9 (improvisation during shared reading) were excluded in subsequent iterations.

The final solution retained eight directly worded items (Items 1, 2, 4, 5, 7, 13, 14, 16) loading on a single factor (Table 2). Standardized loadings ranged from 0.51 to 0.78, the eigenvalue was 3.14, and the factor accounted for 39.2% of the variance. Parallel analysis confirmed unidimensionality. Fit indices were mixed (χ^2^(20) = 118.94, *p* < 0.001; RMSEA = 0.106 [0.088, 0.124]; SRMR = 0.048; TLI = 0.870; CFI = 0.907): SRMR was well below the 0.08 threshold and CFI approached the 0.90 cutoff, but RMSEA exceeded 0.08. This dissociation is consistent with a correctly specified unidimensional model containing localized residual dependencies between content-adjacent items (e.g., items pertaining to the regulation of negative affect through play), and is also expected given that RMSEA is biased upward in models with few degrees of freedom (Kenny et al., 2015). All loadings remained substantive and theoretically interpretable, and further item deletion did not improve fit. The eight-item set was retained for confirmatory testing on the independent subsample.

**Table 2:** Factor loadings for the final 8-item PPQ (exploratory subsample, *n* = 444).

Item	Item (English/Russian translation)	Loading	Uniqueness
13	In times of stress, I try to help my child loosen up with a joyful activity. Если ребенок испытывает стресс, я стараюсь помочь ему расслабиться с помощью какого-нибудь веселого занятия.	0.78	0.39
2	When my child is in a bad mood, I help him or her by asking him/her to play with me or do a fun activity. Когда у ребёнка плохое настроение, чтобы помочь ему, я предлагаю совместную игру или веселое занятие.	0.68	0.53
14	When we go to new places like the zoo or an amusement park, I like to watch my child explore and encourage him/her to ask questions. Когда мы бываем в новых местах (например, в зоопарке или парке развлечений), мне нравится наблюдать, как ребёнок их исследует, и поощрять его задавать вопросы.	0.63	0.60
5	I like to make everyday activities (bath time, brushing teeth) fun by dancing, singing, or playing games with my children. Мне нравится делать рутинные дела с ребенком (например, купание, чистка зубов) весело - танцуя, напевая или играя с ним.	0.62	0.61
7	When my child is afraid of doing something, I look for ways to change the scary situation into a game, a challenge, or something funny. Когда ребенок боится что-то сделать, я ищу способы превратить пугающую ситуацию в игру, состязание или что-то забавное.	0.62	0.62
4	When my child is resistant, I look for ways to make him/her laugh, or to turn the situation into a game, in order to help him/her come out of his/ her oppositional state. Когда ребенок капризничает и упрямится, я ищу способы рассмешить его или превратить ситуацию в игру, чтобы помочь ему выйти из этого состояния.	0.58	0.66
1	As a parent, I find that using games or humor helps me figure out and resolve parenting conflicts. Как родитель, я вижу, что игры и юмор помогают мне справляться и находить решения конфликтов при воспитании ребенка.	0.55	0.70
16	I often use role games or stories to help my child learn new things. Я часто использую ролевые игры или истории, чтобы помочь своему ребенку научиться чему-то новому.	0.51	0.74

Extraction: minimum residuals on polychoric correlations. Eigenvalue = 3.14; variance explained = 39.2%.

### Confirmatory factor analysis for the item-reduced parental playfulness questionnaire (PPQ-8)

To cross-validate the factor structure identified in the exploratory phase, a confirmatory factor analysis (CFA) was performed on the holdout subsample (*n* = 445). Given the ordinal nature of the response scale, non-normality of the item distributions, and observed ceiling effects, the model was estimated using the robust weighted least squares estimator (WLSMV with polychoric correlations and robust standard errors).

The single-factor model demonstrated good fit to the data. Although the scaled chi-square test was statistically significant, χ^2^(20) = 58.94, χ^2^/df ≈ 2.95, *p* < 0.001, all approximate fit indices met or exceeded the conventional thresholds recommended by Hu and Bentler (1999): CFI = 0.976, TLI = 0.967, RMSEA = 0.066, 90% CI [0.047, 0.086], *p*(RMSEA ≤ 0.05) = 0.080, and SRMR = 0.054. Additional indices were also indicative of an acceptable to excellent fit (NFI = 0.965, IFI = 0.976, RNI = 0.976, GFI = 0.995, MFI = 0.983).

All standardized factor loadings were statistically significant (*p* < 0.001) and of acceptable magnitude, ranging from 0.563 (item 1) to 0.795 (item 13), with a mean loading of 0.638. Item-level R^2^ values ranged from 0.317 to 0.632, indicating that the latent Playfulness factor explained a substantial proportion of variance in each indicator. No modification indices exceeded the predefined cutoff, supporting the unidimensional structure without the need for *post hoc* respecification. Taken together, these results provide strong confirmatory evidence for the one-factor structure of the Parental Playfulness Scale.

Internal consistency of the eight-item Parental Playfulness Scale (PPQ-8) was examined using both McDonald’s *ω* and Cronbach’s *α*, with 95% confidence intervals estimated through bootstrap resampling. Both indicated acceptable reliability: ω = 0.790, 95% CI [0.755, 0.818], and α = 0.784, 95% CI [0.748, 0.811]. Item-level analyses showed that the deletion of any single item would not have substantially improved overall reliability; “ω-if-item-dropped” values ranged narrowly from 0.750 to 0.781, and “α-if-item-dropped” values from 0.741 to 0.779, all remaining below or close to the scale-level estimates. These results indicate that all retained items contribute coherently to the measurement of the latent construct and that the scale demonstrates satisfactory internal consistency for research applications.

### External validity of the item-reduced parental playfulness questionnaire (PPQ-8)

Bivariate correlations were used on the confirmatory subsample to examine the external validity of the PPQ-8 (see Table 3). All associations were statistically significant at *p* < 0.001 (Table 3).

**Table 3:** Spearman correlations between the PPQ-8 and external measures.

Variable	1	2	3	4	5
1. PPQ-8	—				
2. CGPQ (Structure)	0.49***	—			
3. Parental warmth	0.46***	0.37***	—		
4. Parental stress	−0.32***	−0.24***	−0.37***	—	
5. Parental satisfaction	0.45***	0.36***	0.52***	−0.44***	—

Spearman’s ρ. *N* ranges from 417 to 445 due to pairwise deletion of missing data. ***p < 0.001.

Additionally, a multiple linear regression was estimated (*n* = 417 after listwise deletion), with the PPQ-8 total score as the dependent variable. The CGPQ Structure total score, parental warmth, parenting stress, and parental satisfaction scores were entered simultaneously as predictors, while child’s gender and age were included as sociodemographic covariates. The model accounted for a substantial proportion of variance in parental playfulness, *F* (6, 410) = 35.30, *p* < 0.001, *R*^2^ = 0.341, adjusted *R*^2^ = 0.331 (Table 4).

**Table 4:** Multiple linear regression predicting parental playfulness (PPQ-8) (*n* = 417).

Predictor	b	SE	t	*p*
(Constant)	6.744	3.191	2.114	0.035
Child’s gender (boy vs. girl)ᵃ	−0.147	0.382	−0.385	0.700
General parenting quality (CGPQ)	0.655	0.073	8.934	< 0.001
Parental warmth	0.351	0.167	2.103	0.036
Parenting stress	−0.149	0.045	−3.309	0.001
Parental satisfaction	0.222	0.094	2.366	0.018
Child’s age	−0.028	0.012	−2.243	0.025

Dependent variable: PPQ-8 total score. R^2^ = 0.341, adjusted R^2^ = 0.331, F (6, 410) = 35.30, *p* < 0.001. b = unstandardized regression coefficient; SE = standard error. ᵃReference category = girl.

The pattern of associations was consistent with theoretical expectations. Parental warmth was positively associated with playfulness, in line with the conceptualization of playfulness as a manifestation of an emotionally warm parenting style. Parenting stress and parental satisfaction emerged as distinct yet meaningful correlates: playfulness was negatively related to stress and positively related to satisfaction, while the magnitude of these associations indicates that playfulness cannot be reduced to the affective-evaluative experience of the parental role. The strongest predictor was the CGPQ Structure total score: parents who are more playful also tend to report higher levels of responsiveness, monitoring, and organization of their child’s daily life, although the two constructs remain clearly distinguishable. Among the covariates, child’s age showed a small negative effect, consistent with developmental evidence that playful parenting is somewhat more prominent during early childhood, whereas child’s gender was unrelated to playfulness.

## Discussion

With respect to the first aim of the study, our initial hypothesis that the original five-factor structure and reliability of the PPQ would replicate in Russian was not supported: four of the five subscales showed unacceptable internal consistency, and a systematic method effect associated with reverse-scored items emerged. However, an empirically derived unidimensional 8-item short form (PPQ-8), composed exclusively of directly worded items, demonstrated good model fit in confirmatory analysis and acceptable internal consistency. With respect to the second aim, the hypothesis was supported: parental playfulness, as measured by the PPQ-8, was negatively associated with parental stress and positively associated with parental satisfaction, parental warmth, and the structural dimensions of general parenting.

The resulting PPQ-8 can be characterized as a unidimensional measure of active, positively expressed parental playfulness. This final solution differs substantially from the original five-factor structure and may be considered a revised instrument rather than simply an abbreviated version. It seems that PPQ-8 should be understood as assessing a specific subtype of parental playfulness rather than the broader multidimensional construct proposed in the original PPQ. All eight retained items describe observable manifestations of playful parenting: using games and humor to resolve conflicts (item 1), transforming everyday routines into joyful activities (items 4, 5), employing playful strategies to regulate the child’s negative affect or fear (items 2, 7, 13), and creatively scaffolding new experiences and learning (items 14, 16). Notably, the item 13. “In times of stress, I try to help my child loosen up with a joyful activity” has the highest factor loading in both EFA and CFA. This finding suggests that in the Russian sample, parental playfulness is anchored in the deliberate use of joyful interaction as a regulatory resource, rather than in spontaneous, aimless fun (Shorer et al., 2021; Magnuson and Barnett, 2013).

In contrast, the items that failed to load on the latent factor were predominantly reverse-scored and aimed at capturing the absence of playfulness (e.g., goal orientation, viewing silliness as a waste of time, irritation with disruptions of plans). Their poor functioning may reflect several co-occurring mechanisms rather than a single source of bias. First, it is consistent with the well-documented reversed-item method bias (Weijters et al., 2013). Second, this bias may be amplified in the Russian cultural context. As anticipated, the dual normative landscape of contemporary Russian parenting may render extreme negative self-descriptions (e.g., “being silly with my child is a waste of time”) highly socially undesirable. This produces pronounced ceiling effects driven jointly by social desirability and a genuinely strong endorsement of involved parenting as a normative imperative (Fedorov and Zlobina, 2025). Although Item 14 also showed a ceiling effect, it was retained in the final solution with a substantive factor loading, which we interpret as reflecting the strong cultural endorsement of supporting children’s curiosity rather than as a threat to its construct validity. Third, the reverse-worded items partially tap substantively distinct facets of playfulness such as the absence of rigidity or goal-fixation that are not merely the inverse of active playful engagement.

Item 11, originally referring to brainstorming Halloween costume ideas, was translated as “New Year and costume parties”. It exhibited the lowest mean among directly worded items, the largest standard deviation, a platykurtic distribution, and a low item–rest correlation. Costume parties are not a salient practice in Russian family life across major holidays, so for many respondents the described situation remained hypothetical, supporting Van de Vijver and Tanzer’s (2004) argument that culturally non-equivalent stimuli undermine measurement validity. Item 19 (“power struggles with my child”) faced an analogous problem at the lexical level: the expression in Russian is strongly associated with political or organizational, rather than family, contexts (Shabanova, 2011). Beyond translation, the framing of parent–child relationships as a “struggle for power” may itself be culturally variable and less salient in contexts where parent–child hierarchy is traditionally taken for granted rather than negotiated (Karabanova, 2005). The exclusion of both items is therefore justified on psychometric, linguistic, and content-validity grounds.

Bivariate and multivariate analyses jointly situate parental playfulness within the broader construct of positive parenting. The Structure dimension of the CGPQ emerged as the strongest predictor of playfulness, showing that playful parenting coexists with (rather than opposes) organization and monitoring. This finding is particularly informative in the Russian context, where structured developmental activities are highly valued (Karabanova, 2005; Polivanova et al., 2024), and supports the conceptual distinctiveness of the two constructs: organizational scaffolding of caregiving and its playful improvisational quality. Parental warmth showed a comparable bivariate association but a more modest unique contribution, replicating Shorer et al. (2021) and confirming that playfulness is related to, yet not reducible to, emotional availability.

A similar interpretation applies to the finding that parental playfulness was negatively associated with stress and positively with satisfaction. This is in line with Russian evidence linking parenting styles to maternal burnout (Apanovich and Serykh, 2024). From an emotion-regulation perspective, playful engagement may help parents reframe stressful interactions, while stress reduces the psychological availability required for play (Shorer et al., 2021). This reciprocal dynamic is also consistent with developmental systems theory (Schneider et al., 2022).

The sociodemographic covariates further qualify the construct. Child’s age showed a small negative effect, indicating that playful engagement was somewhat more prominent with younger children. This fits developmental evidence that improvisational parenting is most salient in early childhood and gradually gives way to other modes of interaction as the child matures (Schneider et al., 2022). Child’s gender, in contrast, was unrelated to playfulness, suggesting that PPQ-8 captures a relatively gender-neutral parental disposition.

Although the adaptation was conducted in Russia, the present findings have broader relevance. First, the PPQ is one of the few instruments designed specifically to assess parental playfulness, and its cross-cultural performance had not previously been examined. Second, the non-replication of the original five-factor structure and the systematic reversed-item method effect raise concerns that may not be specific to the Russian context but rather reflect general vulnerabilities of the PPQ. Future revisions of the instrument may benefit from reconsidering the use of reverse-scored items. Third, by deriving and validating a brief unidimensional 8-item version with good psychometric properties, we offer an efficient instrument suitable for inclusion in large-scale surveys, longitudinal panels, and intervention studies where respondent burden is a critical concern.

Several limitations must be acknowledged. Measurement invariance across parental gender could not be tested due to the strong underrepresentation of fathers (3.49%). Cross-cultural evidence indicates that fathers’ and mothers’ parenting practices may have distinct effects on child outcomes (Quang Dao and Le, 2025), underscoring the importance of testing measurement invariance across parental gender in future research. Consequently, the present findings should be interpreted as reflecting predominantly maternal playful parenting practices, and the generalizability of the PPQ-8 to fathers remains an open empirical question. The sample was self-selected through municipal educational organizations and may overrepresent more engaged and highly educated parents. Test–retest reliability was not assessed, leaving the temporal stability of the PPQ-8 an open question. The cross-sectional design precludes inferences about the direction of the playfulness–stress association, which should be examined in longitudinal designs. Finally, the loss of the five conceptually meaningful subscales (Spontaneity, Problem-Solving, Creativity, Emotion Regulation, Cognitive Flexibility) means that researchers interested in differentiated facets of playfulness must consider whether a unidimensional indicator, focusing on the regulatory function of parental playfulness, is sufficient for their purposes. A further limitation is the absence of a direct measure of social desirability. Although we interpret the ceiling effects and reversed-item method effect as partly reflecting the cultural normativity of involved parenting, we cannot rule out that high scores also reflect socially desirable responding. Finally, the exclusion of all reverse-scored items may have inflated internal consistency while reducing content coverage.

Future research should examine measurement invariance of the PPQ-8 across gender, child age, and cultures, establish its test–retest reliability, and link parental playfulness to observed parent–child interactions and child developmental outcomes. The PPQ-8 may also serve as a practical tool for screening and for evaluating positive parenting interventions. Given the diversity of parenting practices across cultural contexts, understanding the role of playfulness in caregiving represents an important step toward a more comprehensive, culturally informed model of parent–child relationships.

## Data Availability

The raw data supporting the conclusions of this article will be made available by the authors, without undue reservation.
